# Oncologic outcomes and survival of modern surgical approaches for gastric gastrointestinal stromal tumor (GIST)

**DOI:** 10.1007/s00464-024-11152-8

**Published:** 2024-08-23

**Authors:** Hadley D. Freeman, Ross Mudgway, Zachary Tran, Rachael Kim, Sharon S. Lum, Jukes P. Namm, Michael P. O’Leary, Mark E. Reeves, Esther Wu, David Caba Molina

**Affiliations:** Department of Surgery, Loma Linda University Health, Loma Linda, CA USA

**Keywords:** GIST, Minimally invasive surgery, Laparoscopic, Robotic

## Abstract

**Background:**

Studies have demonstrated comparable outcomes between laparoscopic and open resection of gastrointestinal stromal tumor (GIST). We sought to compare outcomes among robotic, laparoscopic, and open resection of gastric GIST in the era of expanding minimally invasive surgery.

**Methods:**

A retrospective analysis was performed of adult patients with gastric GIST undergoing definitive surgery using the National Cancer Database from 2010 to 2020, excluding cases converted to open. Patients were stratified into minimally invasive surgery (MIS), (combined *robotic (R)* and *laparoscopic (L)*), and *open (O).* Hospital length of stay (LOS), 30-day mortality, 90-day mortality, and margin status were assessed. Subgroup analysis was performed to evaluate outcomes between R and L cohorts. Entropy balancing was used to adjust for intergroup differences. Kaplan–Meier survival estimates were used to compare unadjusted 5-year survival.

**Results:**

Of the 15,022 patients (*R* = 10.4%, *L* = 44.3%, *O* = 45.3%), 63.2% were stage I and 70.6% underwent partial gastrectomy. MIS approach was associated with shorter hospital LOS (*β*: − 2.58; 95% CI: − 2.82 to − 2.33) and lower odds of 30-day (OR 0.45; 95% CI: 0.30–0.68) and 90-day mortality (OR 0.54; 95% CI: 0.39–0.74) compared to *O*. Likelihood of R0 resection similar between groups (OR 1.00; 95% CI: 0.88–1.14). Hospital LOS (β: + 0.25; 95% CI: − 0.14–0.64), odds of 30-day (OR 0.99; 95% CI: 0.40–2.46) and 90-day mortality (OR 0.89; 95% CI: 0.47–1.70), and rate of R0 resection (OR 1.02; 95% CI: 0.82–1.27) were comparable between *R* and *L* cohorts. Compared to *O*, MIS approach was associated with improved 5-year OS (log rank *p* < 0.001). Overall survival was not significantly different between *R* and* L* (log rank *p* = 0.44).

**Conclusion:**

These findings suggest that MIS approach may be considered for resection of gastric GIST in select patients. Among patients receiving an MIS approach, the robotic technique can be considered an oncologically safe alternative to laparoscopic surgery.

**Graphical Abstract:**

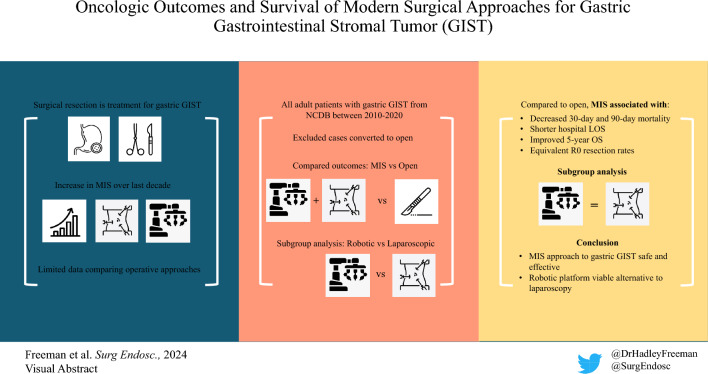

The use of minimally invasive surgery (MIS) for the treatment of gastric gastrointestinal tumors (GIST) has shown increased utilization over the last decade [1, 2]. Furthermore, MIS approach to gastric GIST has been associated with improved perioperative outcomes, preservation of oncologic parameters and comparable survival when compared to the open approach [3]. While there have been concerns regarding MIS resections in patients with large tumors due to the risk of lesion rupture and recurrence, studies have suggested comparable survival between MIS and open approaches to gastric GIST ≥ 10 cm and those receiving neoadjuvant therapy [1].

In addition to laparoscopic surgery, the robotic platform specifically is now being employed more frequently to treat gastrointestinal malignancies [1, 4]. Robotic surgery provides added benefits in terms of maneuverability, ergonomics, dexterity, and three-dimensional visibility [5]. Previous work has shown comparable perioperative outcomes between robotic and laparoscopic approaches to gastric GIST, although it is notable the robotic approach is associated with longer operative times and higher costs [6]. Additionally, a systematic literature review proposed that a robotic approach may be preferable for larger gastric GISTs in unfavorable locations and reserved for more challenging cases [7]. Although recommendations for surgical resection of gastric GISTs are not standardized and data is largely retrospective, MIS treatment for GIST has been shown to be a safe approach for appropriately sized tumors.

As the utilization of MIS, particularly robotic operations, increases for gastrointestinal cancers, it is important that further research be conducted to compare outcomes of surgical approaches. To our knowledge, there are few studies that compare laparoscopic, robotic, and open resection as distinct categories in analyzing gastric GIST resection outcomes. Therefore, we sought to compare outcomes between robotic, laparoscopic, and open resection of gastric GIST using the National Cancer Database.

## Materials and methods

### Study design

**Fig. 1:**
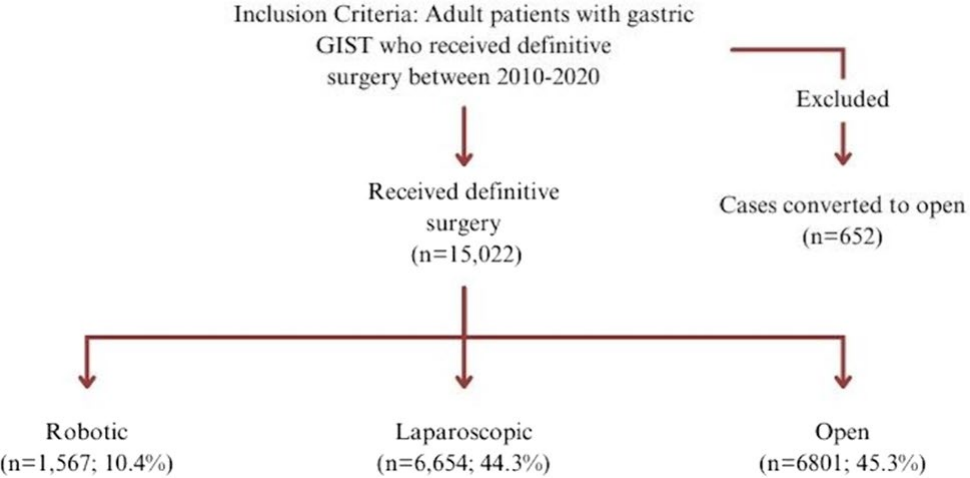
Case selection and inclusion criteria

This retrospective cohort analysis included all adult patients (≥ 18 years) undergoing operations for gastric GIST within the National Cancer Database (NCDB) from 2010 to 2020. The NCDB is a joint project of the Commission on Cancer (CoC) of the American College of Surgeons (ACS) and the American Cancer Society and captures approximately 72% of all cancer diagnoses among ACS-accredited cancer programs [8]. The time period was selected due to 2010 as the first year when surgical approach reporting was mandated in the NCDB. Patients were categorized into 3 surgical approach groups: *Robotic (R)*, *Laparoscopic (L)*, and *Open (O)*. Of note, *L* patients include both trans-abdominal and endoscopic approaches as the NCDB does not differentiate these categories. Minimally invasive (MIS) was further defined to include *R* or* L* approaches. Cases converted to open were excluded. See Fig. 1 for complete case selection criteria.

### Participants

Patient and hospital characteristics were defined in accordance to the NCDB Participant User Files data dictionary [9]. Variables of interest included age, sex, race and ethnicity, insurance status, income quartile, and treatment facility type. Race and ethnicity were identified in the NCDB by medical record and surname [10]. Oncologic variables included tumor grade and size, AJCC pathologic stage, extent of resection, receipt of chemotherapy, and margin status. Quantification of burden of comorbidity was defined by the modified Charlson/Deyo index provided by the NCDB and described elsewhere [11].

The primary endpoint was proportion of microscopically negative (R0) margins. Secondary outcomes included 30-day mortality, 90-day mortality, hospital length of stay (LOS), and 5-year overall survival.

### Statistical methods

Categorical variables were compared using the Chi-square test and reported as proportions. Continuous variables that are not normally distributed, such as age, are reported as medians with interquartile range (IQR) and were analyzed using the Mann–Whitney U test. Trends were analyzed using a rank-based non-parametric test developed by Cuzick (NP-trend) [12]. Multivariable logistic and linear regression was used to evaluate the association of operative approach with outcomes of interest. Elastic Net with retention of clinically relevant variables were used for feature selection [13]. This machine learning-based technique combines ridge regression with least absolute shrinkage and selection operator (LASSO) to select explanatory variables. Kaplan–Meier survival estimates with log-rank test (log rank p) was used to compare unadjusted overall survival between surgical approach groups. Cox proportional hazards were used to evaluate the adjusted hazard of survival and reported as hazard ratios. Patients lost to follow-up or were still alive by study end were censored.

To account for inter-group differences among operative approach, entropy balancing was used [14]. This methodology does not rely on creating propensity scores and therefore allows for the retention of the entire patient cohort for analysis. Regression outcomes are reported as adjusted odds ratios (AOR) and β-coefficient for dichotomous and continuous variables, respectively. Statistical significance is defined as *α* < 0.05. All statistical models were performed using Stata/MP 14.0 (StataCorp, College Station, TX) [15]. Due to the de-identified nature of NCDB data, this study was deemed exempt from formal review by the Loma Linda University Institutional Review Board.

## Results

**Fig. 2:**
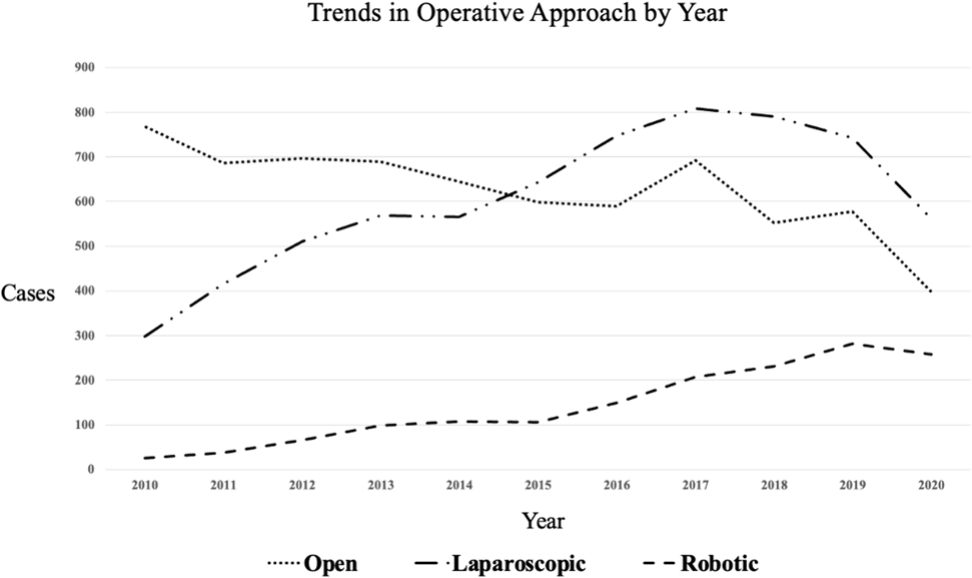
Trends in operative approach for gastric GIST (2010–2020)

Of the 15,022 patients included for analysis, 6801 (45.3%) were *O*, 6654 (44.3%) were *L*, and the remainder (1567, 10.4%) were *R*. Baseline clinicopathologic characteristics are shown in Table 1. Over the study period, the proportion of minimally invasive approaches increased with *R* comprising 20.6% of all GIST operations by 2020 (Fig. 2, NP-trend < 0.001). Compared to *O*, MIS patients were more commonly younger (median age 65 (57–73) years vs. 66 (57–74), *p* = 0.007), female sex (53.7% vs. 51.0%, p = 0.001), White race (68.4% vs. 65.7%, *p* < 0.001), privately insured (40.5% vs. 38.1%, *p* < 0.001), in the highest income quartile (2016–2020) (45.4% vs. 40.8%, *p* < 0.001), and treated at academic programs (42.7% vs. 42.3%, *p* = 0.014). Although Charlson-Deyo score was similar among groups, patients were less frequently Black race (23.6% vs. 26.8%, *p* < 0.001) in the MIS cohort. Regarding oncologic characteristics, MIS patients were more likely to have AJCC stage I disease (73.0% vs. 51.4%, *p* < 0.001), tumors ≤ 2 cm (97.5% vs. 91.7%, *p* < 0.001), and well-differentiated tumors (52.6% vs. 38.1%, *p* < 0.001). MIS patients were more likely to undergo partial gastrectomy (73.0% vs. 67.8%, *p* < 0.001). Receipt of chemotherapy was less likely in MIS patients (19.6% vs. 36.7%, *p* < 0.001), including neoadjuvant therapy (5.8% vs. 11.8%, *p* < 0.001). Time to definitive surgical intervention was longer among MIS (12 (0–53) days vs. 6 (0–45), *p* = 0.014).

**Table 1:** Clinicopathologic characteristics of 15,022 patients with gastric GIST, stratified by treatment type (MIS vs. Open)

	MIS (*R* + *L*) (*n* = 8221, 54.7%)	Open (*n* = 6801, 45.3%)	*p* value
Age (median)	65 (57–73)	66 (57–74)	0.007
Sex			
Female	4418 (53.7)	3469 (51.0)	0.001
Male	3803 (46.3)	3332 (49.0)	
Race			
White	5620 (68.4)	4470 (65.7)	< 0.001
Black	1940 (23.6)	1825 (26.8)	
Asian/Pacific Islander	549 (6.7)	434 (6.4)	
Other/unknown	112 (1.4)	72 (1.1)	
Ethnicity			
Hispanic	517 (6.3)	409 (6.0)	0.486
Non-Hispanic	7704 (93.7)	6392 (94.0)	
Insurance status			
Medicaid	4029 (49.0)	3338 (49.1)	< 0.001
Medicare	491 (6.0)	421 (6.2)	
Private	3326 (40.5)	2593 (38.1)	
Uninsured	90 (1.1)	91 (1.3)	
Other	285 (3.5)	358 (5.3)	
Annual income category			
< $30,000	870 (12.3)	837 (14.5)	< 0.001
$30,000–$34,999	1062 (15.1)	974 (16.9)	
$35,000–$45,999	1924 (27.3)	1609 (27.9)	
$46,000 +	3202 (45.4)	2354 (40.8)	
Facility type			
Community cancer program	348 (4.4)	319 (4.9)	0.014
Comprehensive community cancer program	2717 (34.2)	2120 (32.4)	
Academic or research program	3399 (42.7)	2765 (42.3)	
Integrated network cancer program	1493 (18.8)	1338 (20.5)	
Charlson/Deyo score			
0	5667 (68.9)	4752 (70.0)	0.563
1	1741 (21.2)	1402 (20.6)	
2	523 (6.4)	405 (6.0)	
≥ 3	290 (3.5)	242 (3.6)	
AJCC pathologic stage			
Stage I	6000 (73.0)	3498 (51.4)	< 0.001
Stage II	896 (10.9)	1007 (14.8)	
Stage III	595 (7.2)	1162 (17.1)	
Stage IV	212 (2.6)	589 (8.7)	
Unknown	518 (6.3)	545 (8.0)	
Tumor size			
≤ 2 cm	8018 (97.5)	6237 (91.7)	< 0.001
2–5 cm (including 5)	59 (0.7)	405 (6.0)	
5.1–10 cm (including 10)	20 (0.2)	33 (0.5)	
Unknown	124 (1.5)	126 (1.9)	
Surgical extent			
Local resection	1485 (18.1)	829 (12.2)	< 0.001
Partial gastrectomy	6004 (73.0)	4609 (67.8)	
Total/near total gastrectomy	85 (1.0)	176 (2.6)	
Partial/total gastrectomy with partial esophagectomy	387 (4.7)	410 (6.0)	
Partial/total gastrectomy with resection of other involved organs	183 (2.2)	687 (10.1)	
Surgery not otherwise specified	77 (0.9)	90 (1.3)	
Tumor grade			
Well-differentiated	4322 (52.6)	3588 (38.1)	< 0.001
;Moderately differentiated	867 (10.6)	856 (12.6)	
Poorly/undifferentiated	667 (8.1)	839 (12.3)	
Unknown	2518 (37.0)	2365 (28.8)	
Chemotherapy			
Yes	1612 (19.6)	2498 (36.7)	< 0.001
No	6609 (80.4)	4303 (63.3)	
Neoadjuvant only			
Yes	473 (5.8)	799 (11.8)	< 0.001
No	7748 (94.3)	6002 (88.3)	
Time to surgery (median days)	12 (0–53)	6 (0–45)	0.014

On unadjusted analysis, MIS was associated with lower likelihood of 30-day mortality (0.5% vs. 1.5%, *p* < 0.001) and 90-day mortality (0.9% vs. 2.3%, *p* < 0.001). Rate of R0 resection was significantly increased among MIS compared to *O* (89.8% vs. 88.0%, *p* < 0.001). Hospital LOS was significantly shorter among MIS compared to *O* (2 days (1–4) vs. 5 (4–8), *p* < 0.001). See Table 2.

**Table 2:** Outcomes based on univariate analysis, stratified by treatment type (MIS vs. Open)

	MIS (*R* + *L*) (*n* = 8221, 54.7%)	Open (*n* = 6801, 45.3%)	*p* value
Margin status			
R0	7378 (89.8)	5987 (88.0)	0.010
R1	276 (3.4)	262 (3.9)	
R2	19 (0.2)	20 (0.3)	
Unknown	548 (6.7)	532 (7.8)	
Length of stay (median days)	2 (1–4)	5 (4–8)	< 0.001
30-day mortality			
Yes	42 (0.5)	104 (1.5)	< 0.001
No	8179 (99.5)	6697 (98.5)	
90-day mortality			
Yes	76 (0.9)	155 (2.3)	< 0.001
No	8145 (99.1)	6646 (97.7)	

**Fig. 3:**
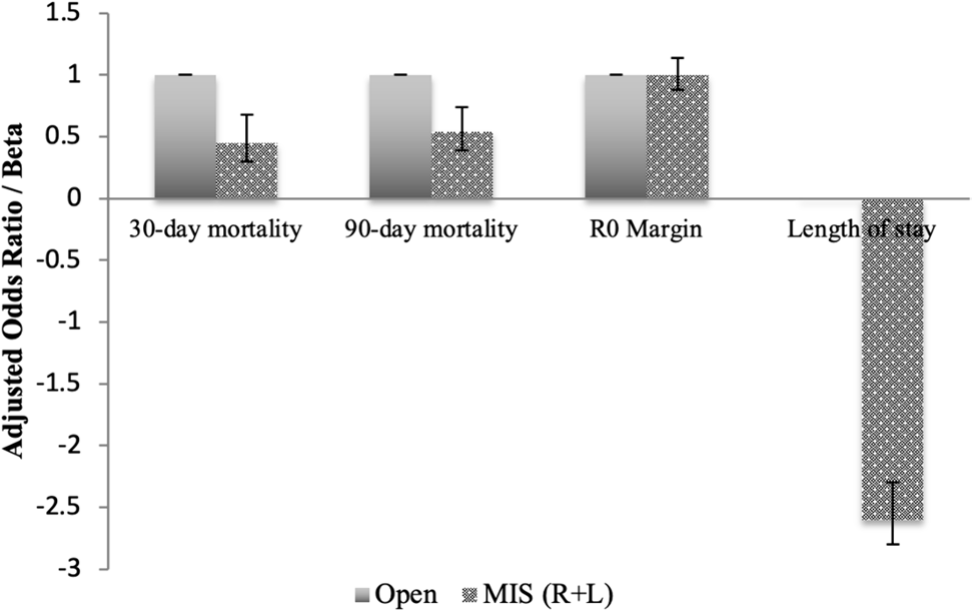
Outcomes based on entropy balancing (MIS vs. Open)

**Fig. 4:**
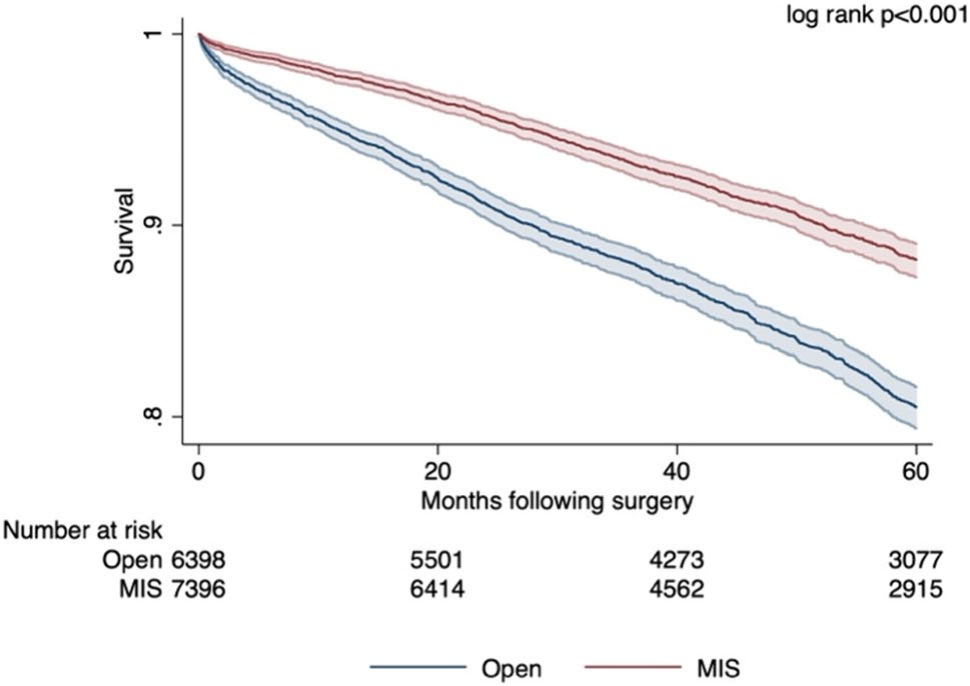
Kaplan–Meier estimated unadjusted 5-year overall survival, MIS versus Open

After adjustment with entropy balancing, MIS was associated with lower 30- (AOR 0.45, 95% CI: 0.30–0.68) and 90-day (AOR 0.54, 95% CI: 0.39–0.74) mortality with *O* as reference. Although odds of R0 resection were similar (AOR 1.00, 95% CI: 0.88–1.14), MIS was associated with a 2.6 day decrement in hospital LOS (95% CI: − 2.8 to − 2.3) (Fig. 3). On survival analysis, MIS was associated with greater 5-year survival compared to *O* (log rank *p* < 0.001) (Fig. 4). After adjustment with Cox proportional hazards analysis, MIS was persistently associated with a lower hazard of 5-year mortality (HR 0.70, 95% CI: 0.63–0.79).

Sub-group analysis was performed comparing *R* and *L* as shown in Table 3 (*n* = 8,221). Compared to *L, R* patients were more likely to be older (median age 66 (58–74) years vs. 65 (56–73), *p* = 0.001), of Hispanic ethnicity (7.7% vs. 6.0%, *p* = 0.013) and have Medicaid insurance (52.0% vs. 48.3%, *p* = 0.001). Furthermore, *R* patients had a greater proportion of AJCC stage I tumors (73.5% vs. 72.9%, *p* = 0.039), tumors ≤ 2 cm (98.5% vs. 97.3%, *p* = 0.010), and well-differentiated tumors (61.1% vs. 50.6%, *p* < 0.001) compared to *L*. Patients undergoing *R* resection were more likely to receive partial gastrectomy (78.0% vs. 71.9%, *p* < 0.001) compared to *L*. Although patient sex, Charlson-Deyo scores, and receipt of chemotherapy were similar between groups, *R* were less likely to be treated at an academic program (41.0% vs. 43.1%, *p* = 0.020) compared to *L*. Time to definitive surgery was significantly longer among *R* (29 (0–66) days vs. 7 (0–49) days, *p* < 0.001).

**Table 3:** Clinicopathologic characteristics of 8,221 patients with gastric GIST, stratified by treatment type (Robotic vs. Laparoscopic)

	Robotic (*n* = 1567, 19.0%)	Laparoscopic (*n* = 6654, 81.0%)	*p* value
Age (median)	66 (58–74)	65 (56–73)	0.001
Sex			
Female	838 (53.5)	3580 (53.8)	0.817
Male	729 (46.5)	3074 (46.2)	
Race			
White	1058 (67.5)	4562 (68.6)	0.041
Black	362 (23.1)	1578 (23.7)	
Asian/Pacific Islander	115 (7.3)	434 (6.5)	
Other/unknown	32 (2.0)	80 (1.2)	
Ethnicity			
Hispanic	120 (7.7)	397 (6.0)	0.013
Non-hispanic	1447 (92.3)	6257 (94.0)	
Insurance status			
Medicaid	814 (52.0)	3215 (48.3)	0.001
Medicare	98 (6.3)	393 (5.9)	
Private	587 (37.5)	2739 (41.2)	
Uninsured	27 (1.7)	63 (1.0)	
Other	41 (2.6)	244 (3.7)	
Annual income category			
< $30,000	169 (12.6)	701 (12.3)	0.218
$30,000–$34,999	224 (16.7)	838 (14.7)	
$35,000–$45,999	365 (27.2)	1559 (27.3)	
$46,000 +	583 (43.5)	2619 (45.8)	
Facility type			
Community cancer program	51 (3.4)	297 (4.6)	0.020
Comprehensive community cancer program	530 (35.0)	2187 (34.0)	
Academic or research program	621 (41.0)	2778 (43.1)	
Integrated network cancer program	314 (20.7)	1179 (18.3)	
Charlson/Deyo score			
0	1076 (68.7)	4591 (69.0)	0.746
1	341 (21.8)	1400 (21.0)	
2	92 (5.9)	431 (6.5)	
≥ 3	58 (3.7)	232 (3.5)	
AJCC pathologic stage			
Stage I	1151 (73.5)	4849 (72.9)	0.039
Stage II	180 (11.5)	716 (10.8)	
Stage III	110 (7.0)	485 (7.3)	
Stage IV	50 (3.2)	162 (2.4)	
Unknown	76 (4.9)	442 (6.6)	
Tumor size			
≤ 2 cm	1543 (98.5)	6475 (97.3)	0.010
2–5 cm (including 5)	2 (0.1)	57 (0.9)	
5.1–10 cm (including 10)	2 (0.1)	18 (0.3)	
Unknown	20 (1.3)	104 (1.6)	
Surgical extent			
Local resection	194 (12.4)	1291 (19.4)	< 0.001
Partial gastrectomy	1222 (78.0)	4782 (71.9)	
Total/near total gastrectomy	20 (1.3)	65 (1.0)	
Partial/total gastrectomy with partial esophagectomy	87 (5.6)	300 (4.5)	
Partial/total gastrectomy with resection of other involved organs	30 (1.9)	153 (2.3)	
Surgery not otherwise specified	14 (0.9)	63 (1.0)	
Tumor grade			
Well differentiated	957 (61.1)	3365 (50.6)	< 0.001
Moderately differentiated	136 (8.7)	731 (11.0)	
Poorly/Undifferentiated	141 (9.0)	526 (7.9)	
Unknown	333 (21.3)	2032 (30.5)	
Chemotherapy			
Yes	317 (20.2)	1295 (19.5)	0.491
No	1250 (79.8)	5359 (80.5)	
Neoadjuvant only			
Yes	70 (4.5)	235 (3.5)	0.078
No	1497 (95.5)	6419 (96.5)	
Time to surgery (median days)	29 (0–66)	7 (0–49)	< 0.001

Unadjusted rates of 30- (0.5% vs. 0.5%, *p* = 0.192) and 90-day mortality (0.8% vs. 1.0%, *p* = 0.663) as well as R0 resection (90.9% vs. 89.5%, *p* = 0.192) were similar, regardless of approach (Table 4). Hospital LOS was longer among *R* (3 (1–4) days vs. 2 (1–4) days, *p* =  < 0.001).

**Table 4:** Outcomes based on univariate analysis, stratified by treatment type (Robotic vs. Laparoscopic)

	Robotic (*n* = 1567, 19.0%)	Laparoscopic (*n* = 6654, 81.0%)	*p* value
Margin status			
R0	1425 (90.9)	5953 (89.5)	0.192
R1	46 (2.9)	230 (3.5)	
R2	1 (0.1)	18 (0.3)	
Unknown	95 (6.1)	453 (6.8)	
Length of stay (median days)	3 (1–4)	2 (1–4)	< 0.001
30-day mortality			
Yes	7 (0.5)	35 (0.5)	0.692
No	1560 (99.5)	6619 (99.5)	
90-day mortality			
Yes	13 (0.8)	63 (1.0)	0.663
No	1554 (99.2)	6591 (99.1)	

**Fig. 5:**
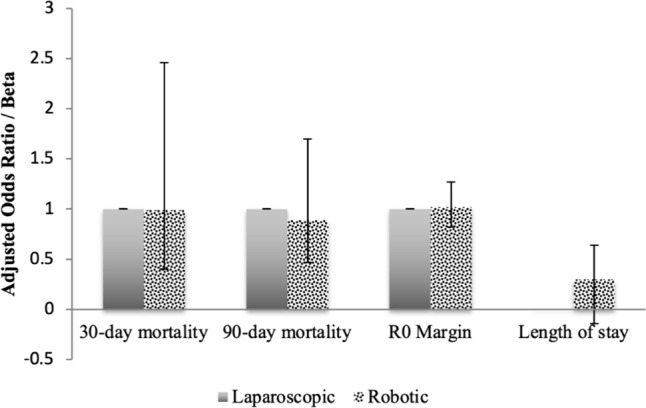
Outcomes based on entropy balancing (Robotic vs. Laparoscopic)

**Fig. 6:**
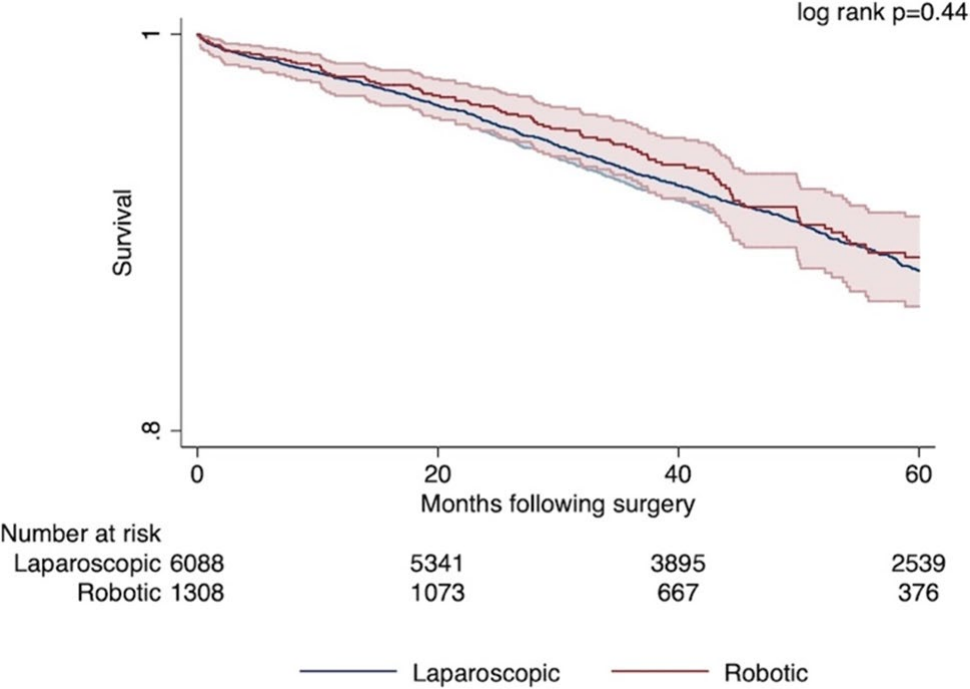
Kaplan–Meier estimated unadjusted 5-year overall survival, Robotic versus Laparoscopic

After adjustment, no significant differences were noted among 30- (AOR 0.99, 95% CI: 0.40–2.46) or 90-day (AOR 0.89, 95% CI: 0.47–1.70) mortality, odds of R0 margin (AOR 1.02, 95% CI: 0.82–1.27), or hospital LOS (*β* + 0.25; 95% CI: − 0.14–0.64) between *R* and* L* cohorts (Fig. 5). Similarly, unadjusted (log rank *p* = 0.44) (Fig. 6) and adjusted 5-year survival were comparable (HR 0.94 95% CI: 0.74–1.19, reference: *L*).

## Discussion

In this NCDB analysis evaluating a large cohort of patients receiving definitive surgery for gastric GIST from 2010 to 2020, there has been an increase in the utilization of MIS approaches, whereas rates of open operations have declined. Compared to open, MIS approach was associated with improved post-operative outcomes and estimated 5-year overall survival, with comparable rates of R0 resection. Outcomes were equivalent between robotic and laparoscopic cohorts. These findings suggest MIS approach is a safe and effective alternative in the treatment of gastric GIST, and furthermore the robotic platform may serve as an equivalent option to laparoscopic surgery in select patients.

Here we describe an increase in the rates of MIS approach to gastric GIST from 2010 to 2020, including both laparoscopic and robotic, with the laparoscopic approach emerging as the most predominant modality at the conclusion of the study period. Conversely, rates of open approach have decreased. These findings are consistent with those reported in other retrospective population-based studies [1, 4]. Work by Gevorkian et al. reported a rise in the proportion of MIS approach to both gastric and small bowel GIST from 2010 to 2016, with a corresponding decline in open approach [1]. Furthermore, in a study by Konstantinidis et al. evaluating national trends in surgical approaches as related to gastrointestinal cancers, the number of MIS (laparoscopic and robotic) resections for esophageal, gastric, pancreatic, colon and rectal cancers increased from 2010 to 2014, while the number of open cases declined [4]. As the application for MIS continues to evolve, these trends in oncologic surgery will likely persist over time.

This study found a higher proportion of patients with AJCC stage I disease and tumors ≤ 2 cm to receive MIS approach, compared to open. Bischof et al. revealed comparable findings, in which smaller tumor size was independently associated with receipt of MIS among gastric GIST patients [3]. Similarly, in a cohort of patients with gastric and small bowel GISTs, patients with smaller tumors (0-5 cm) were more likely to undergo minimally invasive resection [1]. While our study population overall had a disproportionately small number of tumors > 5 cm, and significantly fewer patients with tumors > 2 cm in the MIS cohort, safety and feasibility of MIS in gastric GIST > 5 cm [7, 21], ≥ 10 cm [1], as well as those in unfavorable locations [7], has been reported. Additionally, our study found higher proportion of local resection (wedge) and partial gastrectomy in MIS patients compared to open, which is consistent with existing literature [7]. These findings suggest MIS approach currently is more often reserved for smaller tumors amenable to wedge resection or partial gastrectomy, although the role for minimally invasive techniques may be expanding.

Separate studies have correspondingly demonstrated improved perioperative outcomes [1–3, 16–20] and oncologic safety [1, 16, 19, 20] in MIS approach for gastric GIST. More broadly on the oncologic spectrum, MIS approach has been associated with more favorable short-term outcomes in patients with rectal cancer [22], prostate cancer [23], endometrial cancer [24] and hepatocellular carcinoma [25], among others. Additionally, oncologic feasibility of MIS resection has been described in other cancers as well [4, 22, 26–28]. Therefore, it is unsurprising that these findings traverse the spectrum of gastric GIST, a subtype of neoplasm which, in routine circumstances, does not require extended margins or lymphadenectomy. Furthermore, similar to our findings, survival benefit has been reported with MIS approach in gastric GIST [1, 21] while others have reported comparable survival between MIS and open [3, 16–20]. As these studies are largely retrospective in nature, it is reasonable to conclude survival with MIS approach is non-inferior to open, with the limitations of not carrying out a non-inferiority trial. Whether survival advantage with MIS is related solely to operative approach cannot be determined in the current retrospective report. The open cohort in our study comprised higher proportions of patients with larger (2−10 cm) and more advanced-stage (stage II-IV) tumors as well as more patients receiving neoadjuvant therapy compared to MIS. Consequently, survival results from this study are rather likely multifactorial in nature and retain a degree of selection bias and indeterminate variability, based on a combination of patient selection, tumor-related factors, operative feasibility, as well as surgeon and institution preference.

Lastly, this report suggests equivalency in outcomes and survival between robotic and laparoscopic resection in gastric GIST. While the robotic platform does offer technical advantages, the vast majority of the tumors (> 97% in both *R* and *L* groups) were ≤ 2 cm and treated with partial gastrectomy or local resection. Additionally, fewer than 5% of patients in each the *R* and *L* cohorts received neoadjuvant therapy (commonly given for locally advanced tumors). The fact that these tumors were small and considered up-front resectable contributes to operative feasibility with comparable *R0* resection rates and survival. Although reports have described feasibility of robotic resection in gastric GIST [29–32], one other study provides comparison between robotic and laparoscopic modalities within this subgroup of patients [6]. According to Solaini et al., while robotic approach conferred longer operative time, conversion rates, complication rates, and safety-related factors were similar between robotic and laparoscopic gastric GIST resection [6]. Despite the paucity of comparison in gastric GIST, a meta-analysis by Guerrini et al. found that while robotic approach was associated with fewer surgical complications, margin status, and recurrence rates were comparable to laparoscopic resection in patients with gastric cancer [33]. Furthermore, a study by Nakauchi et al. also described lower complication rates with robotic compared to laparoscopic gastrectomy in patients with gastric cancer, with similar survival between groups [34]. Given these findings, the robotic platform may serve as a favorable surrogate to laparoscopic resection in appropriately selected patients with gastric malignancies.

Importantly, limitations which are inherent to the retrospective nature of this study should be acknowledged. There are likely unmeasured confounding factors relating to our observations on outcomes by surgical approach. Additionally, we excluded all cases converted to open (n = 378, 4.6%; *R* = 0.53%, *L* = 4.14%) which confers a degree of selection bias. Sensitivity analyses with addition of the small number of cases converted to open did not impart any statistical difference in outcomes. While the NCDB is a validated population-based data registry, there remains a proportion of unknown data which was included in our study population (see Table 1 and Table 3). The NCDB data set does not classify specific chemotherapy or drug agents. We presume chemotherapy data from this study is reflective of targeted therapy for GIST, however this is a large assumption and could contribute to selection bias. Despite its clinical relevance, data reflective of mutational analyses was poorly coded within the NCDB from 2010–2020. Among 25,792 patients with gastric GIST in the NCDB from 2010–2020, a total of 74.0% of patients had either “unknown” or “missing” mutation data. We therefore conclude that the addition of mutational analyses to our dataset would not be accurate or truly reflective of its association with outcomes studied, yet consider this to be a potential confounding factor. Furthermore, specific cell type (epithelioid, spindle, mixed) is data which is not captured by the NCDB. Included within the *L* cohort are an unknown number of patients receiving endoscopic resection for gastric GIST. While the exact proportion of endoscopic resections is not available, we surmise these are small, less aggressive tumors which may contribute to both selection bias as well as the improvement in survival as seen in the MIS cohort. Moreover, this data set included year 2020, the first year of the COVID-19 pandemic, during which NCDB data reporting was disrupted [35]. Therefore, it is possible that gastric GIST cases were underreported during this time. Finally, cases of gastric GIST treated at institutions not accredited by the CoC are not represented in this study. Despite these limitations, the NCDB data is a robust and high-quality depiction of national oncological trends, and our study is one of the first to compare the outcomes of laparoscopic, robotic, and open resection as distinct approaches to gastric GIST.

## Conclusion

Within the realm of oncologic surgery, there has been a rise in the adoption of MIS over the last decade. Safety and efficacy of MIS platform has been corroborated. Compared to open resection, laparoscopic, and robotic modalities for gastric GIST are associated with favorable short- and long-term outcomes and sustained oncologic adequacy. Moreover, the robotic approach appears to be a safe alternative to laparoscopy in this setting. Although patient selection is paramount and surgical decisions can be complex in nature, consideration should be given to MIS approach for these tumors.
